# Transcultural Adaptation and Validation of the Spanish Version of the Visual Analogue Scale for the Foot and Ankle (VASFA)

**DOI:** 10.3390/jcm13010213

**Published:** 2023-12-29

**Authors:** Pablo Cervera-Garvi, Maria Hermas Galan-Hurtado, Ana Marchena-Rodriguez, Esther Chicharro-Luna, Cristina Guerra-Marmolejo, Salvador Diaz-Miguel, Ana Belen Ortega-Avila

**Affiliations:** 1 Department of Nursing and Podiatry, Faculty of Health Sciences, Ampliación de Campus de Teatinos, University of Malaga, Arquitecto Francisco Penalosa 3, 29071 Málaga, Spain; pcervera@uma.es (P.C.-G.); mhgalan@uma.es (M.H.G.-H.); cguerra@uma.es (C.G.-M.); salvadordiazm@uma.es (S.D.-M.); anaortavi@uma.es (A.B.O.-A.); 2 Department of Behavioural and Health Sciences, Nursing Area, Faculty of Medicine, Miguel Hernandez University, San Juan de Alicante, 03550 Alicante, Spain; ecluna@umh.es; 3 Biomedical Research Institute (IBIMA), 29590 Malaga, Spain

**Keywords:** cross-cultural adaptation, reliability, scale, validity, function, pain

## Abstract

Background: The main aim of this study is to perform a cross-cultural adaptation and validation of the Visual Analogue Scale for the Foot and Ankle (VASFA) questionnaire, creating a Spanish-language version (VASFA-Sp), and to determine the measurement properties of this instrument. Methods: VASFA was cross-culturally translated into Spanish following the guidelines of the International Society for Pharmacoeconomics and Outcomes Research (ISPOR). The study sample was composed of 228 participants who were recruited from February to May 2022. All were at least 18 years old, gave signed informed consent to take part and properly completed the Foot and Ankle Ability Measures-Sp and VASFA-Sp questionnaires. Cronbach’s alpha and test/re-test reliability values were calculated. Structural validity was assessed via exploratory factor analysis. Results: The 228 patients included in the final analysis presented the following characteristics: 35.53% were male and 64.47% were female; the mean age was 35.95 (18–81) years; and the mean body mass index was 23.79. Internal consistency was excellent. The Cronbach’s alpha for VASFA-Sp was 0.96 and the intraclass correlation coefficient was 0.932 (95% CI; 0.84 to 0.97). Exploratory factor analysis identified one main factor. Conclusions: VASFA-Sp is a reliable, valid and sensitive questionnaire that is suitable for measuring perceived foot and ankle function impairment in a Spanish-speaking population.

## 1. Introduction

Foot and ankle injuries are often accompanied by complications such as pain, instability or stiffness [1]. In response to this, patient-reported outcome measures (PROMs) are commonly used to obtain and evaluate subjective perceptions of the patient’s condition.

Clinicians need specific PROMs to accurately measure or assess foot and ankle function. In this respect, various questionnaires have been used, such as the Identification Foot and Ankle Instability (IdFAI) scale [2] and the Foot Health Status Questionnaire (FHSQ) [3]. However, the Visual Analogue Scale for the Foot and Ankle (VASFA) [4] is the only one that uses three subscales to assess patients’ perceptions of their functional condition and of changes in their health-related quality of life due to foot problems. This self-administered questionnaire is composed of 20 items, with three subscales to assess function, pain and other complaints.

VASFA was cross-culturally adapted to Finnish in 2017 by Repo et al. [5], creating a version that has good psychometric properties and is considered a good measure to quantify foot/ankle function and pain. It has an overall Cronbach’s alpha of 0.88. For the subscales, this value ranges from 0.81 to 0.96. Versions of VASFA have also been validated for use in Thai, Turkish and Indian languages (Malayalam) [6,7,8], with good levels of validity and reliability.

Different cross-cultural adaptations have been validated in Spanish for patients with specific pathologies such as diabetes mellitus [9] or arthritis [10], but there is a need to validate self-administered questionnaires that measure different foot and ankle disorders, level of function, activity of daily living and pain in the same questionnaire [11].

The aim of our study was to perform a cross-cultural adaptation and validation of a Spanish-language version of the VASFA scale, to analyse its measurement properties and assess the reliability and objectivity of the results obtained when this questionnaire is completed by patients and the results analysed by clinicians.

## 2. Materials and Methods

This study was approved by the corresponding Ethics Committee and was carried out in accordance with the Declaration of Helsinki. All participants gave signed, informed consent before taking part.

The measurement properties of VASFA-Sp were evaluated in accordance with the Consensus-Based Standards for the Selection of Health Status Measurement Instruments (COSMIN) recommendations [12].

### 2.1. Participants

The participants were all patients of the University of Malaga (UMA), recruited from February to May 2022. In total, 228 participants met the following inclusion criteria: they were at least 18 years of age, their first language was Spanish, they had a level educational background without being illiterate and were healthy. Patients were excluded if they had undergone surgery of the foot and/or ankle or had history of ankle sprain in the last six months. All were capable of completing the questionnaires without assistance.

### 2.2. Translation and Cross-Cultural Adaptation

**Figure 1:**
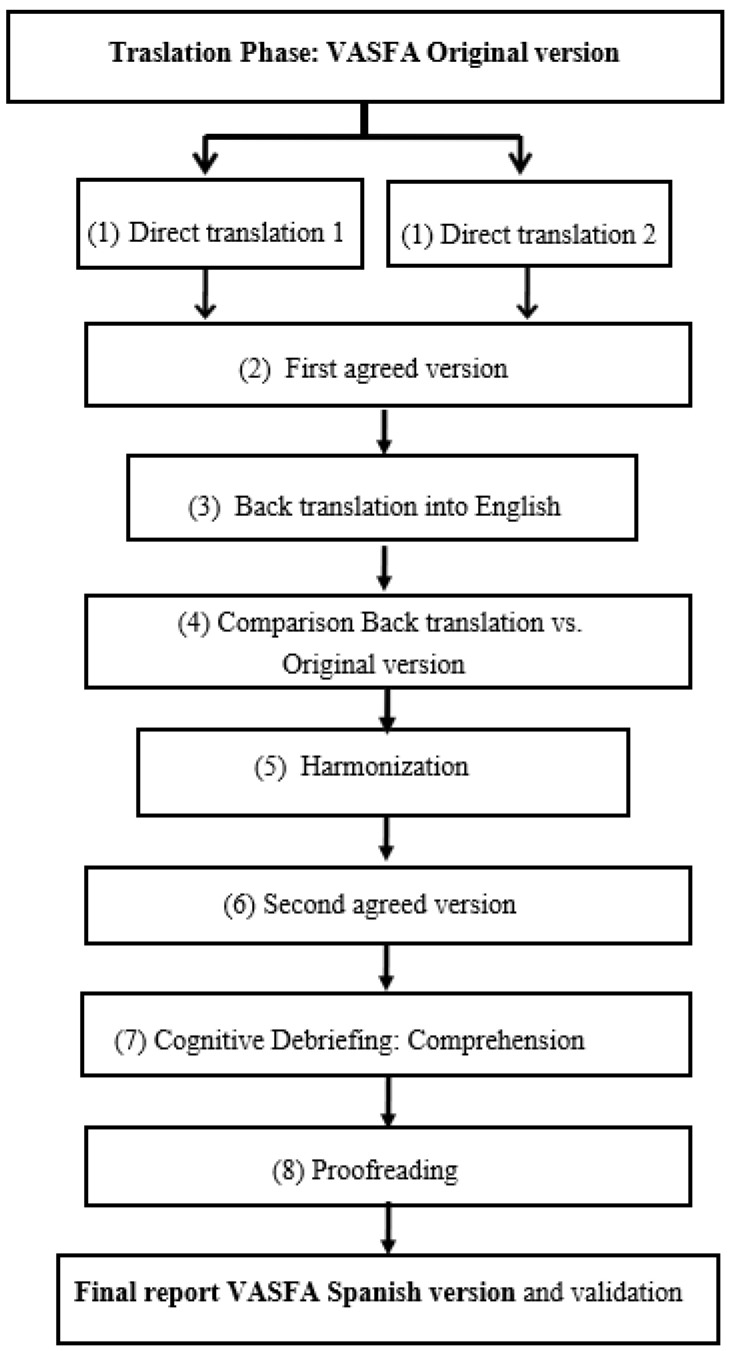
Cross-cultural adaptation process of VASFA-Sp.

The VASFA scale was translated into Spanish following the guidelines of the Patient-Reported Outcomes Measurement Information System (PROMIS) and the International Society for Pharmacoeconomics and Outcomes Research (ISPOR) [13], within eight discrete stages, as follows: (1) The questionnaire was directly translated into Spanish by two bilingual Spanish translators with knowledge of the health sciences, working independently. (2) Their translations were unified by consensus. (3) This version was back-translated into English by two bilingual native English speakers, blinded to the original version. (4) The project leader reviewed the back translation against the source to check for discrepancies. (5) To verify that the translations were complete and correct, a committee of experts, including patients, podiatrists, nurses and translators, reviewed both documents. For greater clarity of the final translated version, any item difficult for participants to understand could be rewritten during this step. (6) This same committee verified the resulting cross-cultural equivalence and developed a final version for field testing. (7) For cognitive debriefing and review, this final version was pre-tested with 15 Spanish patients and any necessary changes made. (8) The project leader checked that there were no errors in either of the translated versions, and corrected any typos observed. Finally, the Flesch Kincaid Grade Level and Flesch Reading Ease tests were performed to assess the readability of the questionnaire. This process of translation and cross-cultural adaptation is illustrated in the flowchart presented in Figure 1.

### 2.3. Data Collection

All participants gave signed consent to take part after being informed of the nature of the study. An independent face-to-face interview was conducted with each patient to obtain the necessary demographic data (gender, age, height, weight, weekly hours of sports activity). All participants completed the Foot and Ankle Ability Measures scale (FAAM) and the Visual Analogue Scale for the Foot and Ankle (VASFA) questionnaires. All confirmed they had no difficulty in understanding the questions.

The FAAM-Sp is a self-administered questionnaire, cross-culturally adapted from the FAAM and validated for use in a Spanish-speaking population, which assesses foot and ankle functions related to the activities of daily living. The Activities of Daily Living (ADL) scale consists of 15 items, scored on a five-point Likert system, ranging from 4 points = No difficulty to 0 points = Unable to perform the action. The total potential score, thus, ranges from 0 to 60 points. This tool has been validated for use with populations presenting various pathologies, including diabetes mellitus, and to also determine the foot and ankle status of elite athletes [14,15]. FAAM-Sp obtained a Cronbach alpha value of 0.95 in a validity and reliability study [16].

VASFA has 20 items grouped into three subscales: function (items 8–17 and 19), pain (items 2–5) and other complaints (items 1, 6, 7, 18 and 20). This instrument consists of 20 questions scored on a visual analogue scale ranging from 0 to 10 points, in which 0 is the worst function score and 10 is the optimal state score. The total potential score of this scale ranges from 0 to 100 points [4].

### 2.4. Statistical Analysis

All statistical analyses were performed with SPSS v.25.0. and SPSS Amos v.26 statistical software (IBM Corporation, Chicago, IL, USA).

#### 2.4.1. Structural Validity

Construct validity was assessed via an exploratory factorial analysis, determining the internal structure and confirming the factors defined in the original version. Bartlett’s test of sphericity was performed to determine whether the factor model was appropriate, and the Kaiser–Meyer–Olkin (KMO) test was performed to assess the sample parameters (very good result considered at ≥0.9).

#### 2.4.2. Discriminant Validity

The cutoff score for VASFA-Sp was assessed according to the receiver operating characteristic (ROC) curve and the area under the curve (AUC) [17].

#### 2.4.3. Test–Retest Validity

The test–retest reliability was evaluated using intraclass correlation coefficients (ICC) [1,2]. VASFA-Sp was administered twice to 20 patients at an interval of seven days. ICC > 0.7 was considered “excellent”, 0.60–0.69, “good”, 0.40–0.59, “fair” and <0.40, “poor” [18].

#### 2.4.4. Internal Consistency

Internal consistency was assessed using Cronbach’s alpha. A value of 0.7 was considered “fair”, and values of 0.8 and 0.9 were considered “good” and “excellent”, respectively, for both the tool and its subscales.

#### 2.4.5. Convergent Validity

Convergent validity was measured with Pearson’s correlations between the VASFA-Sp and FAAM-Sp. Coefficients < 0.30 were considered indicative of poor correlation, <0.70 moderate correlation and ≥0.70 strong correlation.

## 3. Results

Of the initial study population of 240 patients, 12 did not fully complete VASFA-Sp and were excluded. Thus, 228 were selected for the final analysis. Of these participants, 35.53% were male and 64.47% were female. The patients’ mean age was 35.95 (18–81) years and the mean body mass index was 23.79. On average, each one performed 4.17 h of physical activity per week (Table 1).

### 3.1. Readability 

On average, the participants required 8–10 min to complete VASFA-Sp, without help from the administrator. The following readability scores were obtained: Flesch Reading Ease test, 44.2; Flesch–Kincaid Grade Level, 9.6. 

The questionnaire obtained a score of 21.3% on the Flesch Reading Ease Test, meaning it was easy to understand for persons aged 20 years or more.

### 3.2. Structural Validity

Exploratory factor analysis showed the correlation matrix to be appropriate, according to the following test results: Kaiser-Meyer-Olkin, 0.935; Bartlett’s test of sphericity, 190 (*p* < 0.001). 

**Figure 2:**
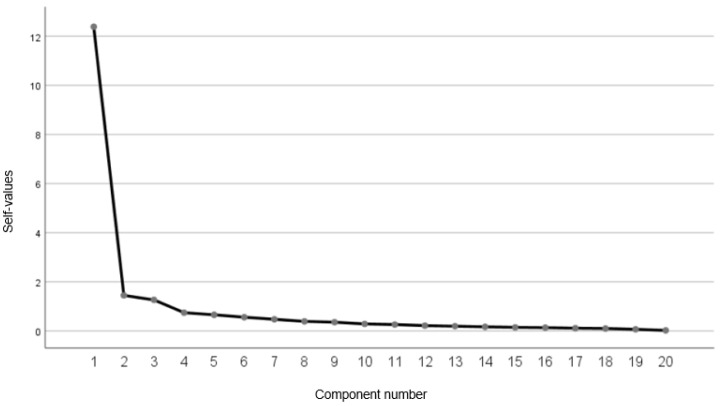
Scree plot.

As shown in the scree plot (Figure 2), a one-factor solution was obtained including the 20 items, which explained 75.49% of the total variance.

### 3.3. Discriminatory Power

**Figure 3:**
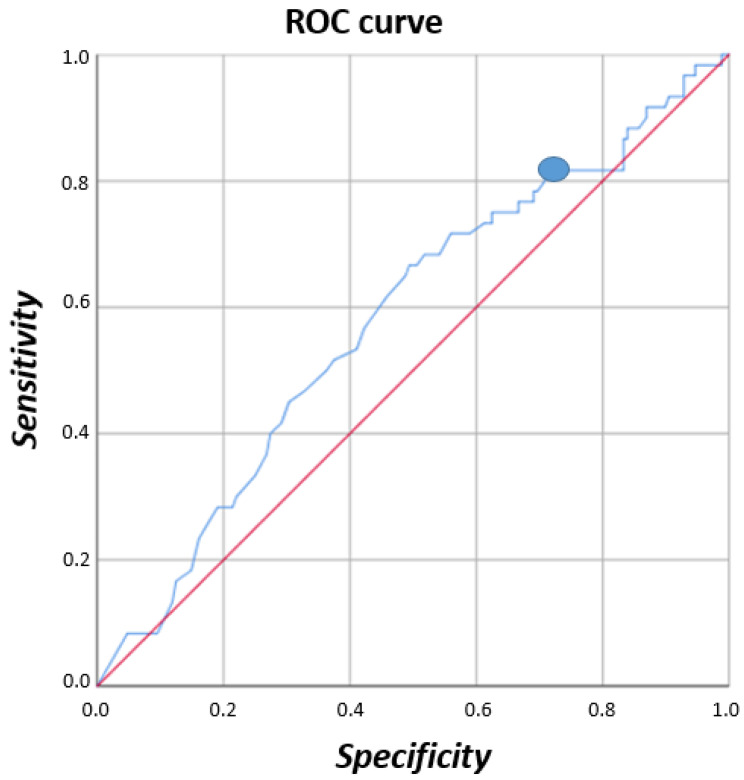
ROC curve.

According to the ROC curve, the model obtained a discrimination score of ≥95 points to identify patients with decreased function (area under the curve, AUC = 0.81, *p* < 0.001) (Figure 3). The optimal cut-off value assumed for the Youden Index was 180. Questionnaire result values >180 reflected a decrease in the patients’ function related to foot and ankle problems, worsening the activity of daily living.

### 3.4. Test–Retest Validity

VASFA-Sp had good test–retest reliability, with a global intraclass correlation coefficient (ICC) of 0.932 (95% CI; 0.84 to 0.97). The standard error of the mean (SEM) was 2.69 and the minimal clinically important difference (MCID) 8.11.

### 3.5. Internal Consistency

VASFA-Sp showed excellent internal consistency, with a Cronbach’s alpha of 0.965. The Cronbach’s alpha values for the subscales were Function = 0.949, Pain = 0.928 and Other complaints = 0.841.

### 3.6. Convergent Validity

Good values were obtained for the correlation between VASFA-Sp and the Spanish version of the FAAM ADL, with a Pearson’s coefficient of 0.583; *p* < 0.001, and the Spanish version of the FAAM Sport, with a Pearson’s coefficient of 0.580; *p* < 0.001.

## 4. Discussion

The aim of this study was to perform a cross-cultural adaption and validation of VASFA to produce a Spanish-language version (VASFA-Sp) and determine its measurement properties, thus providing clinicians in Spanish-speaking environments with a useful instrument to assess patients’ functional capabilities.

The cross-cultural adaptation process was carried out without any relevant problems. Of the participants, 95% answered all the questions and items of the administered questionnaires. During the adaptation, there were no readability problems, and no words or phrases were changed. Data collection was carried out in a room isolated from the clinics to maintain the confidentiality and anonymity of the included participants.

Our analysis of VASFA-Sp included patient’s functional capacity for activities of daily living and for physical activity. The dimension of pain was also considered as a factor that has a direct, negative impact on the patient. Our results confirm that the Spanish version of the questionnaire is reliable and valid.

The study sample was composed of 228 persons, with a mean age similar to that obtained in studies of other versions of VASFA. However, the proportion of women among our participants was lower, although this value is not strictly comparable, since the sample populations in earlier research were much smaller. Thus, the studies of the Finnish [5] and Turkish [7] versions of VASFA had sample sizes of 165 and 128 patients, respectively, while the study of the Thai version [6] had only 42. 

The educational level was only assessed in the Finnish version [5] and in our study. The majority of participants had a high level of education. None of our participants reported any comprehension difficulties or other problems in reading or answering the questionnaire, which was completed in 8–10 min, in line with the results for the Malayalam version [8], for which participants took an average of 9 min to complete. In addition, we carried out a reading and comprehension analysis, from which it was concluded that the questionnaire is of moderate difficulty and can be administered without problems of understandability to any patient over 20 years of age. This analysis was not performed on any of the versions considered in previous studies.

Regarding the psychometric properties of VASFA-Sp, our results corroborate its validity and reliability, with a one-factor construct that accounts for 75.49% of total variance in contrast to the Finnish version, for which two factors were identified with a variance of 70%. The optimal cut-off value assumed for the Youden Index was 0.18. Thus, questionnaire results > 180 reflect decreased physical function.

VASFA-Sp presented excellent internal consistency, with a Cronbach’s alpha of 0.965. This value is similar to that obtained for other adaptations, such as the Finnish version (0.95) and the Turkish version (0.96, with respect to healthy patients). However, the Thai version was even better in this regard, with a Cronbach’s alpha of 0.995.

The reliability of our instrument was confirmed by a test–retest, performed seven days after the first administration. By comparison, the test–retest of the Turkish version was performed after five days, and that of the Finnish version after 14 days. The ICC value of our version was 0.93, demonstrating its good reliability, in line with other adaptations.

Regarding the ceiling–floor effect, our results indicated that there was no ceiling–floor effect in the overall scores or its subscales a maximum of 3%. This is consistent with the Finnish version [5]. It is worth noting that the sample size in our study was significantly higher than in the other versions. Additionally, none of the items reached the maximum limit.

The instruments used to assess the convergent validity of different versions of VASFA vary widely, including questionnaires and scales such as the American Orthopedic Foot and Ankle Society (AOFAS) [19], the Short-Form 36 (SF-36) [20], the Lower Extremity Functional Scale (LEFS) [21], the Foot Function Index (FFI) [22], the Hannover Questionnaire (Q) [23] or the Foot and Ankle Outcome Score (FAOS) [24]. The correlations with the original VASFA include 0.34 with the SF-36 for the Turkish version and 0.7 with the Q for the original version. The highest correlation was obtained with the LEFS, for the Finnish version (r = 0.84). In the present study, we chose the FAAM because of its similarity to the VASFA, because it is specific to foot and ankle disorders and, moreover, because it analyses functions related to the activities of daily living. The Pearson correlation value obtained (0.58) was good and could be further improved if the sample population were composed only of patients with a foot pathology, as was the case for the Malayalam and Finnish versions.

Our study presents certain limitations. The gender difference among the participants means that the study sample is not totally homogeneous should be controlled in future studies, including more participants in the group with a lower participation percentage. Furthermore, individuals with a prior history of pain in the foot and ankle were excluded, and the majority of them had a high level of education, which could have influenced the obtained results. On the other hand, this may also be considered an important strength since psychosocial factors are known to be associated with female gender and influence the patient’s perceptions regarding musculoskeletal pain and activities of daily living [25]. Another possible limitation is the fact that our participants were recruited from private podiatry clinics. This factor may have biased our results since these patients normally present specific alterations such as ankle instability or undiagnosed pathologies. Furthermore, the Rasch analysis might usefully have been performed to re-examine the internal structure. An important strength of our approach is the implementation of a rigorous methodology to analyse the validity of the VASFA-Sp questionnaire for persons with foot and ankle disorders. Finally, future studies should seek to analyse the population by different age groups to better assess the reliability of the measurement instrument used and validate the instrument in populations with specific pathologies such as diabetes mellitus to adequately measure the possible decrease in daily activity influenced by their disease.

## 5. Conclusions

VASFA-Sp is a reliable, valid and sensitive questionnaire to measure perceptions of foot and ankle function impairment in a Spanish-speaking population. The application of the questionnaire can reveal the patient’s degree of foot and ankle dysfunction over time. Accordingly, it can be considered a useful clinical instrument to assess the decrease in the quality of life and function of patients with pathologies or during convalescence processes.

## Figures and Tables

**Table 1:** Characteristics of participants.

Sample Size		(*n* = 228)
Gender	Male Female	81 (35.53%) 147 (64.47%)
Level Education	High level Low level	221 (96.93%) 7 (3.07%)
	Mean	SD
Age	35.95	(15.71)
Height	168.16	(9.04)
Weight	67.59	(14.00)
BMI	23.79	(3.79)
VASFA-Sp	165	(40)
FAAM-ADL	55	(6)

BMI: body mass index; VASFA-Sp: Visual Analogue Scale for the Foot and Ankle-Spanish; FAAM: Foot and Ankle Ability Measures; ADL: Activities of Daily Living.

## Data Availability

The data presented in this study are available on request from the corresponding author.
